# Localization of natriuretic peptide receptors A, B, and C in healthy and diseased mouse kidneys

**DOI:** 10.1007/s00424-022-02774-9

**Published:** 2022-12-08

**Authors:** Elena-Sofia Heinl, Katharina Anna-Elisabeth Broeker, Claudia Lehrmann, Rosmarie Heydn, Katharina Krieger, Katharina Ortmaier, Philipp Tauber, Frank Schweda

**Affiliations:** Institute for Physiology, University Regensburg, Regensburg, Germany

**Keywords:** Natriuretic peptide, Natriuretic peptide receptor, Nephroprotection, ANP, BNP, CNP

## Abstract

The natriuretic peptides (NPs) ANP (atrial natriuretic peptide) and BNP (B-type natriuretic peptide) mediate their widespread effects by activating the natriuretic peptide receptor-A (NPR-A), while C-type natriuretic peptide (CNP) acts via natriuretic peptide receptor-B (NPR-B). NPs are removed from the circulation by internalization via the natriuretic peptide clearance receptor natriuretic peptide receptor-C (NPR-C). In addition to their well-known functions, for instance on blood pressure, all three NPs confer significant cardioprotection and renoprotection. Since neither the NP-mediated renal functions nor the renal target cells of renoprotection are completely understood, we performed systematic localization studies of NP receptors using in situ hybridization (RNAscope) in mouse kidneys. NPR-A mRNA is highly expressed in glomeruli (mainly podocytes), renal arterioles, endothelial cells of peritubular capillaries, and PDGFR-receptor β positive (PDGFR-β) interstitial cells. No NPR-A mRNA was detected by RNAscope in the tubular system. In contrast, NPR-B expression is highest in proximal tubules. NPR-C is located in glomeruli (mainly podocytes), in endothelial cells and PDGFR-β positive cells. To test for a possible regulation of NPRs in kidney diseases, their distribution was studied in adenine nephropathy. Signal intensity of NPR-A and NPR-B mRNA was reduced while their spatial distribution was unaltered compared with healthy kidneys. In contrast, NPR-C mRNA signal was markedly enhanced in cell clusters of myofibroblasts in fibrotic areas of adenine kidneys. In conclusion, the primary renal targets of ANP and BNP are glomerular, vascular, and interstitial cells but not the tubular compartment, while the CNP receptor NPR-B is highly expressed in proximal tubules. Further studies are needed to clarify the function and interplay of this specific receptor expression pattern.

**Supplementary information:**

The online version contains supplementary material available at 10.1007/s00424-022-02774-9.

## Introduction

The group of natriuretic peptides (NPs) includes the structurally related hormone atrial (ANP), B-type (BNP), and C-type natriuretic peptide (CNP) that possess various cardiovascular, renal, and endocrine effects. ANP and BNP are circulating heart-derived peptides, secreted in response to wall stress in the atrium and ventricles, whereas CNP acts in a paracrine manner and, in the cardiovascular system, is produced mainly in the endothelium [33]. ANP, CNP, and urodilatin, which is an alternative cleavage product of the ANP precursor, are also synthesized in the kidney itself, presumably forming locally acting NP systems [40, 57, 69]. Besides their crucial role in maintaining blood pressure, NPs also have strong protective potential, especially with regard to the cardiovascular system and the kidney [8, 27, 41].

NPs exert their biological actions by binding to their specific natriuretic peptide receptors (NPR). While ANP and BNP are ligands for natriuretic peptide receptor-A (NPR-A), CNP acts via natriuretic peptide receptor-B (NPR-B). NPR-A and NPR-B are membrane-bound guanylyl cyclases (guanylyl cyclase-A and -B, respectively), and their activation results in the formation of cyclic guanosine monophosphate (cGMP) [33]. Since, as discussed below, activation of NPR-B by CNP does not induce natriuresis but plays a fundamental role in bone formation and blood pressure regulation, the nomenclature NPR-B, for “natriuretic” peptide receptor, can be misleading and the use of “guanylyl cyclase B” (GC-B) instead of NPR-B has been suggested [1]. In line, the term “guanylyl cyclase A (GC-A)” can be used instead of “NPR-A”. The receptor natriuretic peptide receptor-C (NPR-C) lacks guanylyl cyclase-activity and acts mainly as clearance receptor for NPs [39]. In addition, recent data suggest that NPR-C is coupled to inhibitory G proteins hereby mediating some of the cardiovascular and metabolic effects of CNP [42, 48]. Due to the widespread distribution of NPRs in various tissues and cell types, the biological effects of NPs are very complex. In the kidney, activation of NPR-A increases glomerular filtration rate (GFR) and renal plasma flow (RPF), induces diuresis and natriuresis, and suppresses renin release [2, 4, 17, 32, 33, 35, 38, 64]. CNP does not have significant diuretic and natriuretic properties and its renal effects are less clear [33]. While the suppression of the renin-angiotensin system and the hypotensive effects most likely contribute to cardiorenal protection by NPs, also, a direct interaction with cardiomyocytes or with components of the immune system has been described [11, 24, 71]. NPs ameliorated renal injury in aldosterone- and high-salt-induced hypertension models, and the beneficial effects appeared to be independent of their antihypertensive mode of action [46]. In addition, NPs confer renoprotection in acute renal failure after ischemia/reperfusion, inflammatory renal injury, and diabetic nephropathy [9, 11, 13, 28, 37, 65]. In line, genetic deletion of NPR-A results in higher levels of proinflammatory cytokines, fibrosis, and mesangial proliferation in the kidney [14, 21, 47]. Whether this anti-inflammatory effect is due to a direct effect on immune cells or mediated indirectly, e.g., via inhibition of the renin system, is unclear.

Despite of profound effects of NPs in the control of renal function and on renal integrity, the underlying mechanisms or involved cell types are only incompletely understood. This is particularly true for the contribution of individual NPR-expressing cell types to the complex regulation of renal function in vivo. Cell type-specific deletion of individual components of the NP system helped to fill this knowledge gap partially. For instance, using podocyte-specific NPR-A knockout mice, we have shown previously that NPR-A signaling in podocytes is dispensable for the effects of ANP and BNP on GFR and natriuresis, but, unexpectedly, exerts direct renoprotection [64]. An essential prerequisite for a better understanding of the renal effects of NPs is knowledge of the exact localization of their receptors. Accordingly, studies on the spatial distribution of NPRs in the kidney have already been performed. However, because these studies either did not include all three NPRs, focused only on individual components of the kidney, or did not allow precise assignment to individual cell types, a detailed and comprehensive overview of the distribution of NPRs is lacking to date [19, 51, 59]. We therefore examined the mRNA expression of NPR-A, NPR-B, and NPR-C using high-resolution RNAscope technology. Our studies extend and complement a recent work on the localization of NPRs in human kidney [19], as the use of murine kidneys allows optimal sample preparation and sufficient material for studies on the exact cellular assignment of the receptors. To identify potential target cells for the renoprotective effects of natriuretic peptides, the distribution of NPRs in diseased kidneys was also investigated. Since NPs have anti-inflammatory properties and their renoprotective effects might therefore be related to direct effects on immune cells [14, 21, 47], the disease model chosen was the adenine nephropathy model characterized by severe tubulointerstitial inflammation and fibrosis.

## Methods

### Animals

Male and female wildtype mice on a C57BL/6 background were used and had free access to standard rodent chow and tap water. All animal experiments were conducted according to Guidelines for the Care and Use of Laboratory Animals published by the US National Institutes of Health and approved by the local authorities (Regierung von Unterfranken, 55.2.2–2532.2–1107).

### Adenine-induced nephropathy

Male mice aged 16 to 22 weeks were fed 0.2% adenine containing diet. Control animals received standard rodent chow. After 3 weeks of treatment mice were anesthetized with buprenorphine (0.05 mg/kg BW), xylazine (10 mg/kg BW) and ketamine (80 mg/kg BW) and kidneys and adrenal glands were perfusion-fixed with 4% paraformaldehyde via the abdominal aorta.

Excess intake of adenine results in the formation of 2,8 hydroxyadenine, which is excreted via the kidneys. Due to its low solubility, 2,8 hydroxyadenine precipitates, resulting in the deposition of hydroxyadenine crystals in the tubular lumen and the renal interstitium, in tubulointerstitial inflammation, fibrosis, and progressive deterioration of kidney function with a three- to fourfold increase in serum creatinine after 3 weeks [67].

### In situ* hybridization using RNAscope*

Detection of mRNA in mouse kidneys was performed using either the RNAscope® Multiplex Fluorescent v2 kit or the chromogenic RNAscope® 2.5 HD Reagent Kit-BROWN (Advanced Cell Diagnostics ACD, Cat. No 322300/323100), according to the manufacturer’s protocol. Fixed kidneys and adrenal glands were dehydrated in concentration ascending ethanol series and embedded in paraffin. Hybridization protocol was performed on 5 μm tissue sections, pre-treated for 15 min with 1 × target retrieval solution and 30 min with protease plus reagent (contained in RNAscope kit). If in situ hybridization was followed by immunofluorescence staining, protease plus treatment was reduced to 20 min. Multiplex RNAscope signals were detected using the AKOYA biosciences Opal™ fluorophores Opal690 (FP1497001KT), Opal570 (FP1488001KT), and Opal Polaris 480 (FP1500001KT) diluted in Tyramide Signal Amplification (TSA) buffer (1:750 for Cy3 and Cy5, 1:500 for Cy2). Slices were mounted with ProLong™ Gold Antifade Mountant mounting medium (Thermo Fisher Scientific) and stored at 4 °C. Negative controls were routinely enclosed and evaluated.

The RNAscope probes used are listed in Table1 below.

**Table 1:** RNAscope probes used for in-situ hybridization

RNAscope® probe	Target	Cat. no (ACD Bio)
Mm-Npr1	Natriuretic peptide receptor A (NPR-A/GC-A)	484531
Mm-Npr2	Natriuretic peptide receptor B (NPR-B/GC-B)	438261
Mm-Npr3	Natriuretic peptide receptor C (NPR-C)	502991
Mm-Nphs1-C2	Nephrin	433571-C2
Mm-Pecam1-C3	Platelet and endothelial cell adhesion molecule 1 (CD31)	316721-C3
Mm-Pdgfrb-C2	Platelet-derived growth factor receptor beta (PDGFR-β)	411381-C2
Mm-renin-C2	Renin	433461-C2
Mm-Epo-C2	Erythropoetin	315501-C2
Mm-Adgre1-C2	Adhesion G protein-coupled receptor E1 / F4/80	460651-C2
Mm-Ptprc-C2	Protein tyrosine phosphatase, receptor type C (CD45)	318651-C2
Mm-Cx3cr1-C2	CX3C motif chemokine receptor (fractalkine receptor)	318651-C2
Mm-Acta2-C2	Actin alpha 2 (alpha-smooth muscle actin)	319531-C2
Mm-Sglt1-C3	Sodium/glucose cotransporter 1 (SGLT1)	468881-C3
Negative Control Probe- DapB	4-Hydroxy-tetrahydrodipicolinate reductase (E. coli)	310043

### In situ* hybridization followed by immunofluorescence staining*

In situ hybridization was performed as described above. Before mounting, slices were washed with PBS, blocked for 30 min with 1% BSA and 10% horse serum solution and incubated with primary antibodies diluted in blocking solution overnight at 4 °C. Slices were incubated with fluorescent-labelled secondary antibodies and 4′,6-diamidino-2-phenylindole (DAPI) for 90 min at room temperature and mounted with Dako Mounting medium. Slides were stored at 4 °C until further use. Antibodies are listed in Table 2.

**Table 2:** Primary and secondary antibodies used for immunofluorescence staining following in situ hybridization

Antibody	Staining	Manufacturer	Dilution
Goat anti-mouse AQP2	RNAscope brown NPR-B kidney	Santa Cruz (sc-9882)	1:200
Mouse anti-mouse megalin	RNAscope brown NPR-B/RNAscope multiplex NPR-B kidney	Santa Cruz (sc-515772)	1:200/1:100
Mouse anti-mouse calbindin	RNAscope brown NPR-B kidney	Swant (D28K)	1:500
Rabbit anti-mouse NKCC2	RNAscope brown NPR-B kidney	Merckmillipore (AB3562P)	1:200
Rabbit anti-mouse CYP11B2 (aldosterone synthase)	RNAscope multiplex NPRs adrenal gland	generously provided by Dr. Gomez-Sanchez [76]	1:100
Donkey anti-mouse TRITC	RNAscope brown NPR-B kidney	Jackson ImmunoResearch Europe Ltd. (715–025-150)	1:400
Donkey anti-goat Cy2	RNAscope brown NPR-B kidney	Jackson ImmunoResearch Europe Ltd. (705–485-147)	1:400
Donkey anti-goat Alexa Fluor™ Plus 647	RNAscope brown NPR-B kidney	Invitrogen (A32795)	1:400
Donkey anti-mouse Cy5	RNAscope brown NPR-B kidney	Jackson ImmunoResearch Europe Ltd. (715–175-151)	1:400
Donkey anti-mouse Cy2	RNAscope multiplex NPR-B kidney	Jackson ImmunoResearch Europe Ltd. (715–485-150)	1:400
Donkey anti-rabbit Alexa Fluor™ Plus 488	RNAscope multiplex NPR-B kidney / NPR-A adrenal gland	Invitrogen (A32790)	1:400
Donkey anti-rabbit Alexa Fluor™ Plus 647	RNAscope multiplex NPR-B and -C adrenal gland	Invitrogen (A32795)	1:400

### Microdissection of single tubular segments

Microdissection was performed on four female and male C57BL/6 wildtype mice. The different nephron segments were identified by their morphology and manually isolated from perfused mouse kidney following a well-established protocol [74]. Briefly, the kidneys were perfused with 10 ml incubation solution (140 mmol/l NaCl, 0.4 mmol/l KH_2_PO_4_, 1.6 mmol/l K_2_HPO_4_, 1 mmol/l MgSO_4_, 10 mmol/l sodium acetate, 1 mmol/l α-ketoglutaric acid, 1.3 mmol/l calcium gluconate, 37.5 mg/100 ml glycine, 48 mg/100 ml trypsin inhibitor, pH 7.4 at 37 °C) containing 0.1 mg/ ml collagenase II (Sigma Aldrich, Cat. No. C6885). Sorting solution was prepared by adding 1 mg/ml PVA 4–88 (Sigma Aldrich) to the incubation solution. After perfusion, one kidney was removed, decapsulated, cut into slices, and transferred into prewarmed collagenase-containing incubation solution in a thermo shaker (Eppendorf; 850 rpm, 10 min, 37 °C). After adding prewarmed incubation solution, 1 ml of the digested tubule solution was transferred into a fresh tube. The contained glomeruli and tubular segments were washed and resuspended in 2 ml of sorting solution, and subsequent sorting of glomeruli and tubules was performed at 4 °C using a dissection microscope. Fresh tubule suspension was taken every 5 min from the digestion solution. 300 glomeruli and 100 of each nephron segment (PT S1/S2, PT S3, loops of Henle, CD) were collected. Since whole kidneys were used in the isolation procedure, no discrimination between cortical and medullary collecting ducts was possible. After collection, samples were centrifuged (5 min, 8,500 rpm, 4 °C) and tubule pellets stored at − 80 °C until further use.

### Isolation of podocytes and glomerular cells

Podocytes and other glomerular cells were isolated from reporter mice with mG/mT expression as described previously [64]. These mice express green fluorescent GFP exclusively in podocytes while all other cells, including all non-podocyte cells in glomeruli, show red fluorescence. In brief, mice were sacrificed by cervical dislocation and 15 ml magnetic bead suspension (37 °C, 2 × 10^6^ beads/ml; Invitrogen, Carlsbad, CA) was infused at a pressure of 100 mmHg into the renal arteries via the abdominal aorta. Subsequently, kidneys were further processed (digestion, cell strainer) and glomeruli were separated from the tissue suspension by a magnet. Glomeruli were further processed to receive a single cell suspension and podocytes (green fluorescence) and all other glomerular cells (red fluorescence, termed “non-podocytes” in the following) were sorted by FACS. On average, 250,000 podocytes were isolated from each mouse. To test for a possible enrichment of NPR mRNA in podocytes or glomerular non-podocytes compared with total kidney, complete kidneys were harvested from six mG/mT reporter mice and mRNA was isolated from them, as described below.

### RNA isolation and cDNA-synthesis

RNA from microdissected nephron segments, isolated podocytes and glomerular non-podocytes were isolated using the Zymo *Quick*-RNA® Microprep Kit (Zymo Research, Cat. No. R1050). Cell lysis was performed by repetitive freezing/unfreezing of the samples in lysis puffer (provided in the kit). Subsequent isolation of the RNA was performed according to the manufacturer's protocol. 200 ng RNA of glomeruli and tubular segments or 100 ng RNA of podocytes and glomerular non-podocytes was used for cDNA synthesis. Reverse transcription reaction was conducted using the FastGene® Scriptase Basic cDNA Kit (Nippon Genetics) with Oligo dT-primers.

Total RNA of mG/mT reporter mouse kidneys, control, and adenine-nephropathy mouse kidneys was isolated with innuSOLV RNA Reagent (IST innuscreen GmbH). One complete kidney per animal was used in each case. Reverse transcription reaction was carried out using 1 µg of RNA and the moloney murine leukemia virus reverse transcriptase (Thermo Fisher Scientific) for control and adenine kidneys. For comparability with podocytes, 100 ng of RNA was used for reverse transcription of RNA from the mG/mT reporter mice (FastGene® Scriptase Basic cDNA Kit, Nippon Genetics).

### Semi-quantitative real-time-PCR

MRNA expression in different nephron segments, podocytes, and kidneys was determined by real-time PCR using SYBR Green (Roche Diagnostics). For relative quantification, a standard curve was constructed by plotting the threshold cycle values (Ct) against serial dilutions of a standard sample consisting of pooled individual samples from the respective experiment. Subsequently, the Ct values of each sample were referenced to the standard curve to determine its expression relative to the pooled standard sample. All values were related to the housekeeper gene RPL32. Primers are listed in Table 3.

**Table 3:** Real-time PCR primers used for relative quantification of NPR mRNA expression

Target gene		Sequence (5′–3′)
RPL32	Sense	tggaggtgctgctgatgtg
	Antisense	cgttgggattggtgactctga
NPR-A	Sense	ctcaacatcacagtaaatcacc
	Antisense	ggctttgcccaaacacatcc
NPR-B	Sense	ttgccaacaccggtcactt
	Antisense	gctccgatgaagcgagtaaga
NPR-C	Sense	ggctcaatgaggaggattacgtg
	Antisense	cacagagaagtccccataccg
α-SMA	Sense	gaagagctacgaactgcctga
	Antisense	tttcgtggatgcccgctg
col1a1	Sense	ctgacgcatggccaagaaga
	Antisense	atacctcgggtttccacgtc
Fibronectin	Sense	aggttcgggaagaggttgtg
	Antisense	ggcgtaatgggaaaccgtgt

### Statistics

Statistical analysis and graphs were generated with GraphPad Prism6. All results are presented as mean values ± standard error of the mean (SEM). Data were tested for normal distribution and homoscedasticity (homogeneity of variance); significances were calculated by *t*-test or Welch test accordingly. *P* < 0.05 was considered significant. Statistical significance was marked according to the following scheme: *p* < 0.05 (*), *p* < 0.01 (**), and *p* < 0.001 (***).

## Results

### Expression of NPR-A in murine kidneys

**Fig. 1:**
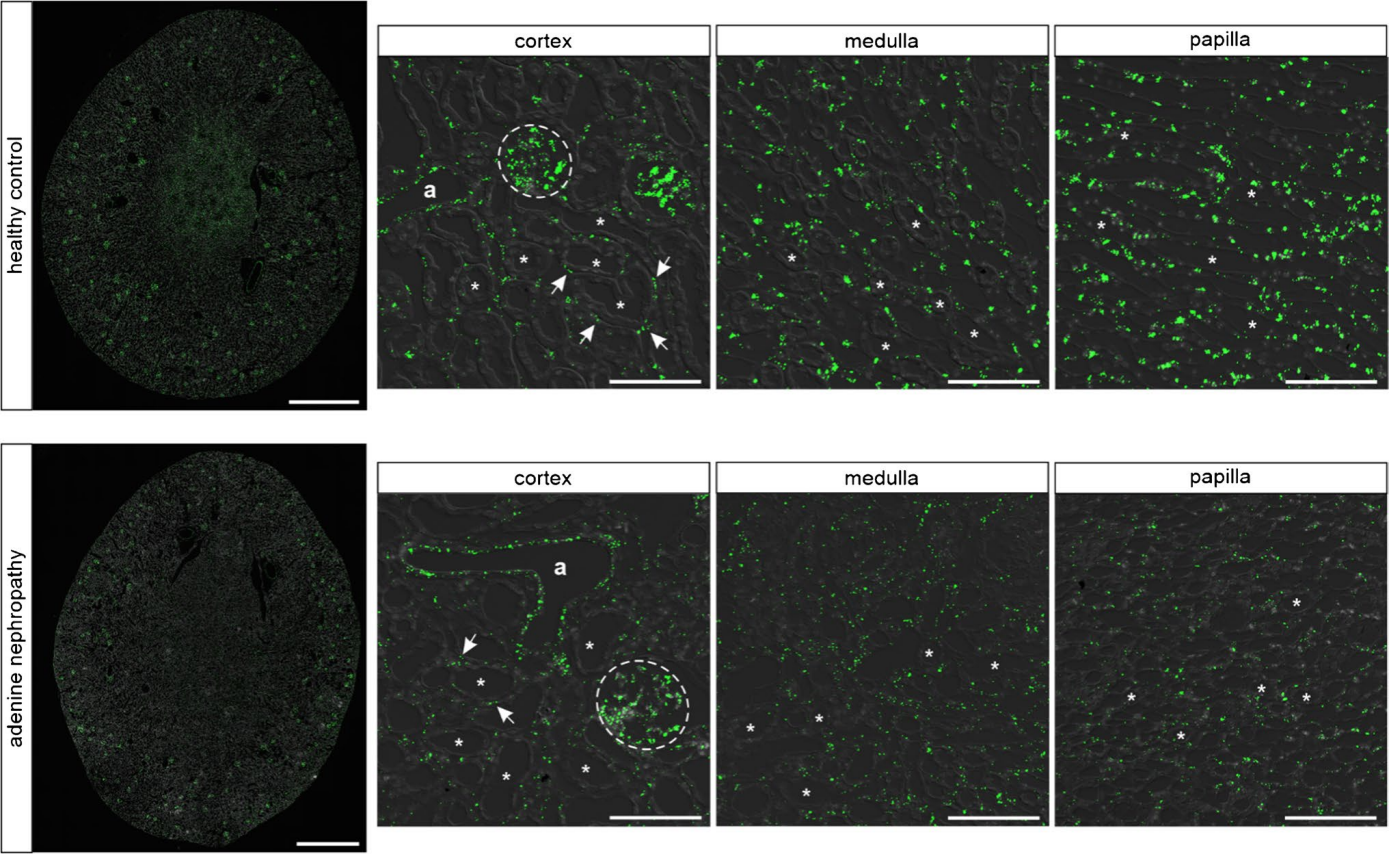
NPR-A expression in murine kidneys under physiological conditions and adenine nephropathy. In healthy controls NPR-A mRNA (green) is mainly expressed in glomeruli (dashed lines), renal arterioles (marked as “a”) and interstitial cells (marked with arrows). No mRNA expression was detectable in tubules (marked as “*”). Interstitial NPR-A expression increased from renal cortex to papilla with the strongest RNAscope signal from cells localized in renal papilla. In adenine nephropathy, a reduction of NPR-A mRNA signal was detectable, particularly in the medullary and papillary areas. Nuclei are counterstained with DAPI (grey). Overview scale bars are 1000 µm. Detail scale bars are 100 µm

The NPs ANP and BNP mediate their biological effects by activating the Natriuretic Peptide Receptor A (NPR-A). NPR-A mRNA was mainly detected in glomeruli, blood vessels, and interstitial cells, with a clear increase in staining intensity from cortex to papilla. Unexpectedly, no NPR-A signal was visible in the tubular system (Fig. 1). Since the distribution pattern of NPR-A expression was not different between male (Fig. 1) and female (online supplement Fig. 2) mice, the following investigations were performed in male mice only.

**Fig. 2:**
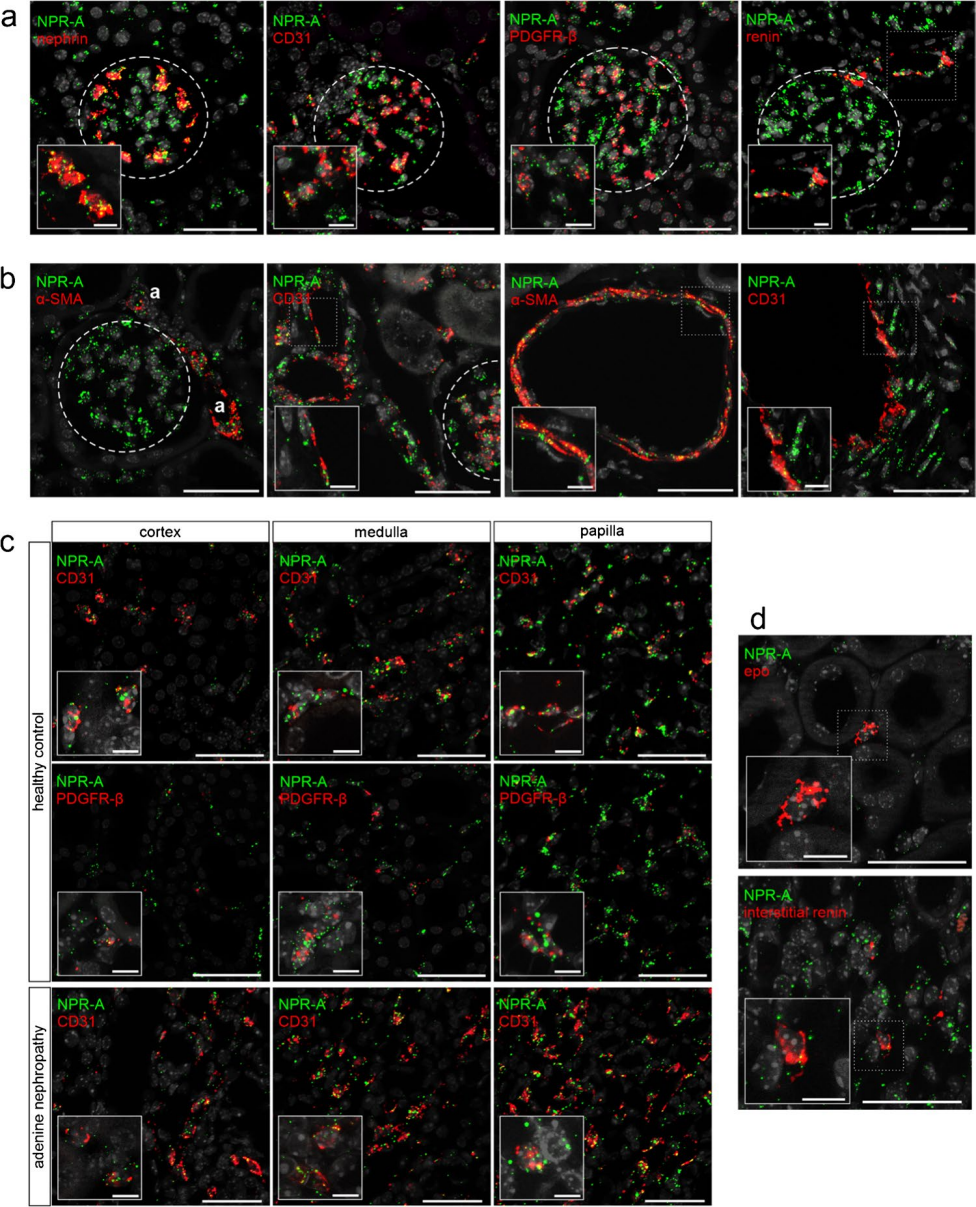
Identification of NPR-A expressing cell types in murine kidneys. mRNA co-localization studies of NPR-A (green) and various cell markers (red) using co-hybridization **a** NPR-A mRNA expression in glomeruli. Colocalization of NPR-A with nephrin (podocytes), CD31 (endothelial cells), PDGFR-β (mesangial cells), and renin (juxtaglomerular renin-producing cells). **b** NPR-A expression in intrarenal arterioles of all sizes, including afferent and efferent arterioles (marked as “a”). Vascular NPR-A mRNA signals predominantly derive from vascular smooth muscle cells (α-SMA). Only few endothelial cells (CD31) showed receptor expression. **c** Extraglomerular NPR-A expression. The majority of extraglomerular NPR-A signal derived from endothelial cells. PDGFR-β^+^ interstitial cells express NPR-A, mostly in renal papilla. Also, in adenine nephropathy, renal NPR-A mRNA signal is mainly assigned to endothelial cells of capillaries. **d** Co-localization of NPR-A and interstitial renin or epo mRNA signals. Nuclei are counterstained with DAPI (grey). Glomeruli are visualized by white dashed lines. Scale bars at lower magnification are 50 µm, at higher magnification 10 µm

In glomeruli, which showed a striking expression of NPR-A mRNA, podocytes were identified as the main NPR-A expressing cell type by duplex RNAscope with nephrin (Fig. 2a). In addition to podocytes, intraglomerular endothelial (co-hybridization with CD31) and mesangial cells (co-hybridization with platelet derived growth factor receptor β, PDGFR-β, a marker for glomerular mesangial cells and interstitial cells) expressed NPR-A (Fig. 2a). Moreover, juxtaglomerular renin-producing cells at the vascular pole of the glomeruli expressed NPR-A (Fig. 2a). Since ANP not only suppresses renal renin release but also the formation of aldosterone in adrenal glands, expression of NPR-A in the adrenal cortex was investigated. In fact, NPR-A is expressed in all cortical zones of adrenal glands including aldosterone-producing cells (online supplement Fig. 3). Intrarenal arterioles of all sizes, including afferent and efferent arterioles, showed clear NPR-A mRNA signals (Fig. 2b). In arterioles, NPR-A signals were predominantly assigned to vascular smooth muscle cells (co-hybridization with alpha smooth muscle actin, α-SMA), whereas endothelial cells (co-hybridization with CD31) had only low NPR-A signal (Fig. 2b). In contrast, endothelial cells of peritubular capillaries and vasa recta showed strong NPR-A signals and were the major extraglomerular cell type expressing NPR-A (Fig. 2c). Additionally, sporadic expression of the receptor was found in cortical PDGFR-β interstitial cells. In the medulla, most signals were assigned to peritubular endothelial cells, but also PDGFR-β cells showed NPR-A expression. In the papilla, NPR-A mRNA was highly detectable in endothelial and PDGFR-β cells. The proportion of PDGFR-β cells that expressed NPR-A continuously increased from the cortex to the papilla, where NPR-A was detectable in almost all PDGFR-β positive interstitial cells (Fig. 2c). The group of PDGFR-β positive interstitial cells contains pericytes, fibroblasts, erythropoietin (epo)-producing cells, but also interstitial cells that express renin [3]. Co-hybridization with epo or renin showed that both cell types expressed NPR-A, but to a lesser extent than endothelial cells (Fig. 2d).

**Fig. 3:**
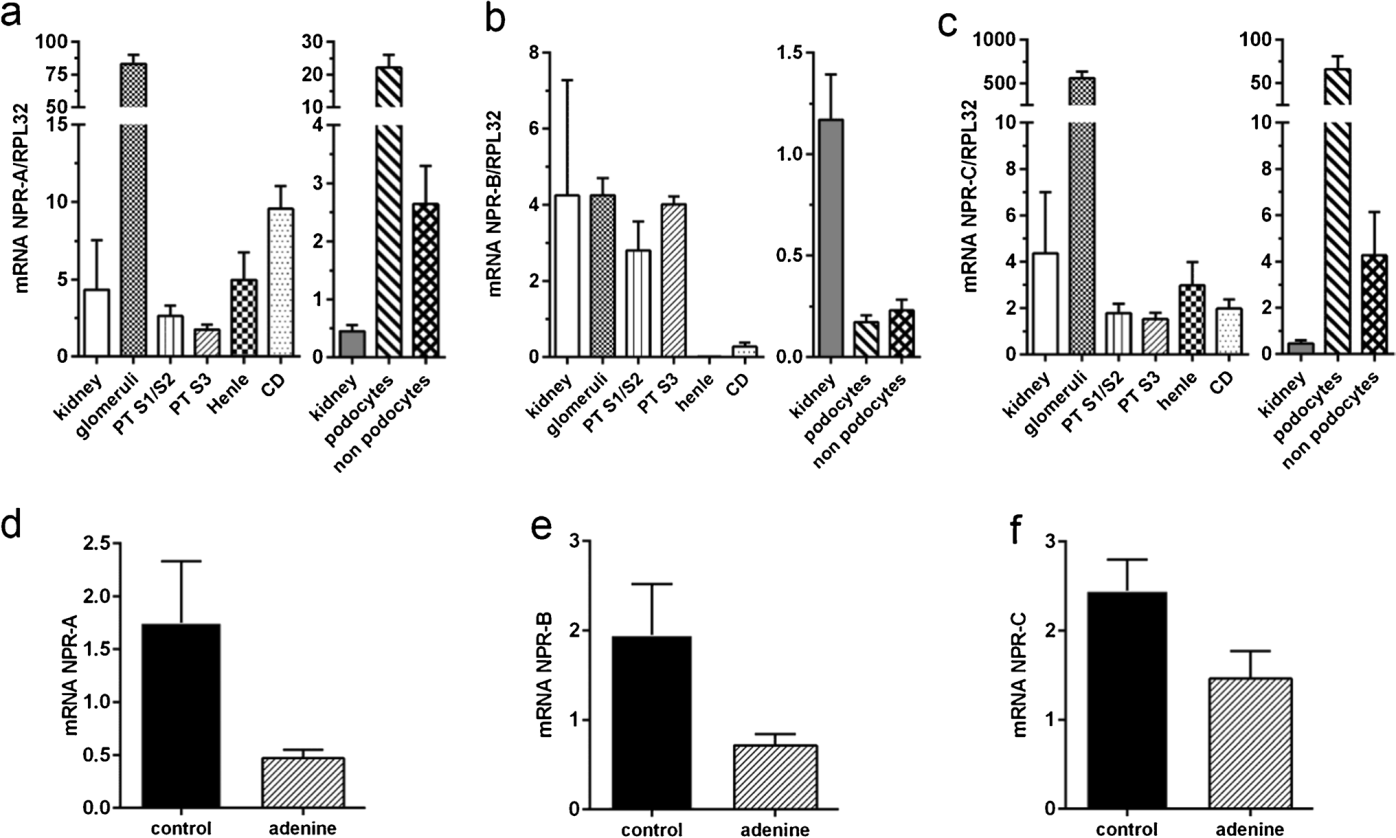
Realtime PCR of NPRs in microdissected tubule segments, glomerular cells, and whole kidneys and in adenine nephropathy. Quantitative analysis of NPR-A, NPR-B, and NPR-C mRNA expression. **a**–**c** Left graphs: mRNA abundance in microdissected nephron segments of healthy C57Bl/6 wildtype mice. For comparison, mRNA expression in whole kidneys of C57Bl/6 wildtype mice is shown. Right graphs: Isolated podocytes and glomerular non-podocyte cells (separation by FACS) of mG/mT reporter mouse kidneys. For comparison, mRNA expression in whole kidneys of mG/mT reporter mice is shown. **d**–**f** NPR mRNA expression in healthy and adenine kidneys. In adenine kidneys, mRNA expression of all three receptors was numerically reduced, without reaching statistical significance. n (control) = 4, n (adenine) = 7. Abbreviations: PT, proximal tubule; CD, collecting duct

Real-time PCR of microdissected nephron segments and isolated podocytes confirmed that NPR-A mRNA was highly expressed in glomeruli and especially in podocytes (Fig. 3a). In contrast to the RNAscope data, NPR-A was detectable in microdissected tubular segments, especially in the collecting duct (CD), however at very low expression levels.

Mice with adenine-nephropathy exhibited severe tubulointerstitial damage, morphologically detectable by fibrotic areas and massive immune cell infiltration, exemplified in the supplemental Online Resource 1a by the leukocyte marker CD45. NPR-A distribution in the kidney was unaltered compared with healthy controls, but signal intensity was markedly decreased especially in the medulla and in the papilla (Fig. 1). The trend of decreasing NPR-A expression in adenine animals was confirmed by realtime PCR in whole kidney homogenates (Fig. 3d). In order to examine NPR-A expression in inflammatory cells of injured kidneys, we performed co-hybridization with immune cell markers (Online Resource 1b). However, no robust and reproducible NPR-A mRNA expression was observed in CD45 (pan leucocyte marker), Cx3cr1 (fractalkine receptor, dendritic cells, macrophages, monocytes), or F4/80 (macrophages) positive cells (see Online Resource 1b) [72]. Besides immune cell infiltrates, adenine-nephropathy is characterized by α-SMA expressing, collagen-producing interstitial myofibroblasts. While few myofibroblasts showed a faint NPR-A signal, most of these cells were NPR-A negative (Online Resource 1b). As in kidneys of healthy mice, by far the largest proportion of NPR-A expression could be mapped to endothelial cells (Fig. 2c).

In summary, in murine kidneys, NPR-A mRNA is expressed by podocytes, mesangial cells, and endothelial cells in glomeruli and by PDGFR-β-positive cells in the interstitium. In renal arterioles, NPR-A is predominantly detectable in vascular smooth muscle cells and less in endothelial cells. In contrast, endothelial cells in peritubular capillaries and vasa recta have high NPR-A expression levels. In mice with adenine nephropathy, marked reduction of NPR-A signal was observed (Table 4).

**Table 4:** Overview of renal cell types expressing NPR-A mRNA in RNAscope in murine kidney

Glomeruli				Interstitium			Tubules	Vessels		Inflammation (adenine nephropathy)			
Capillaries (CD31)	Mesangial cells (PDGFR-β)	Podocytes (nephrin)	JG renin cells	PDGFR-ß interstitial cells	Endothelial cells (CD31)	Renin-/epo-producing cells	All segments	Smooth muscle cells	Endo-thelium (CD31)	Leukocytes (CD45)	Macro-phages (F4/80)	Dendritic cells (Cx3cr1)	Myo-fibroblasts (α-SMA)
+	** + **	** + + **	** + **	** + **	** + + **	** + **	**-**	** + + **	** + **	**-**	**-**	**-**	**-**

Abbreviations: JG: juxtaglomerular, epo: erythropoietin

Symbols representing expression levels: - = no expression. + = low expression. ++ = high expression.

### Expression of NPR-B in murine kidneys

**Fig. 4:**
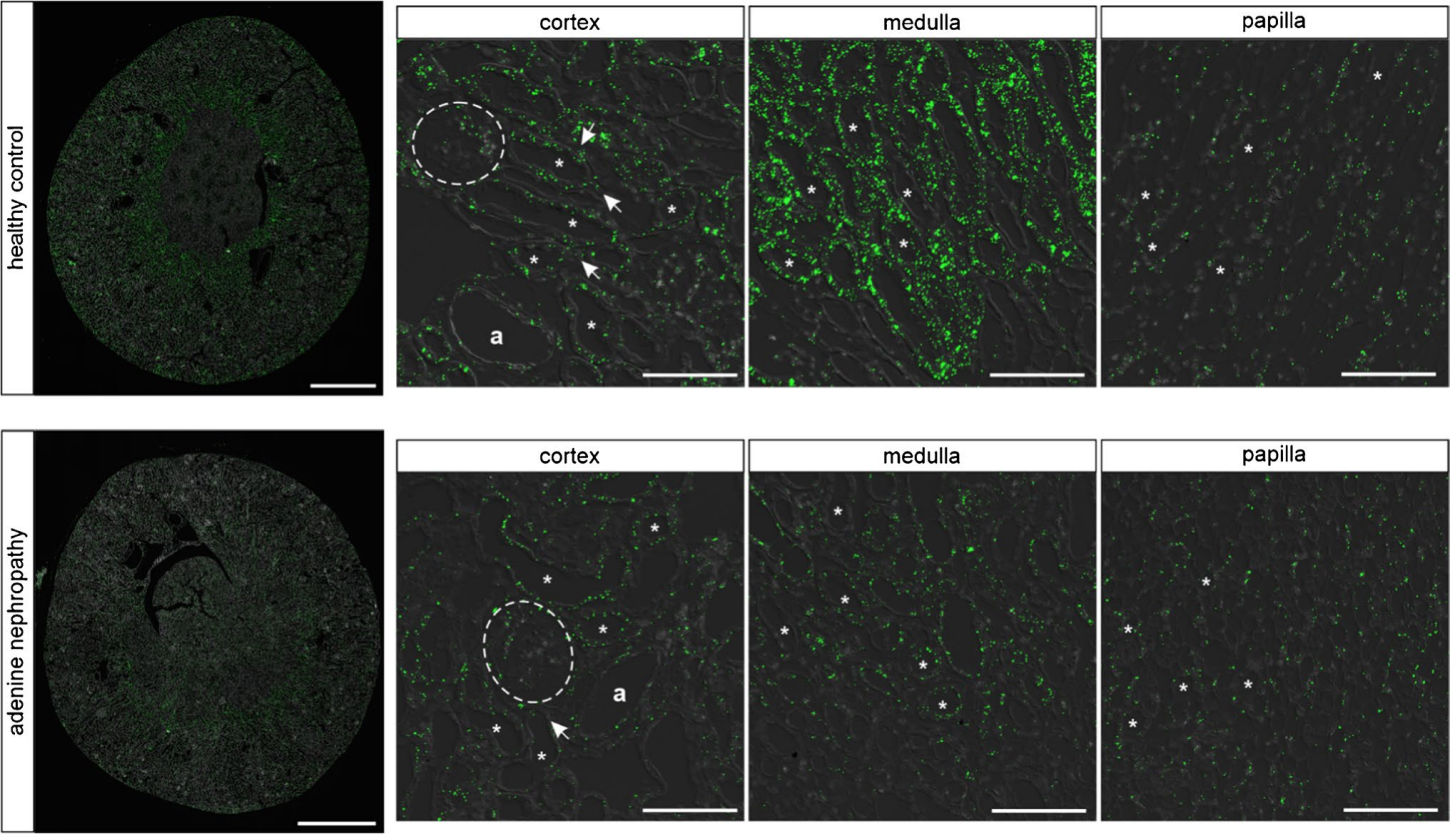
NPR-B expression in murine kidneys of control animals and in adenine nephropathy. NPR-B mRNA distribution (green) in healthy mouse kidneys and in adenine nephropathy. The NPR-B receptor is expressed in the cortical and medullary tubular system (marked as “*”). At a lower level, mRNA signal was found in interstitial cells and in glomeruli (white dashed lines). In adenine nephropathy NPR-B mRNA signal was reduced, while distribution pattern remained the same as in healthy controls. Renal arterioles are marked as “a.” Nuclei are counterstained with DAPI (grey). Overview scale bars are 1000 µm. Detail scale bars are 100 µm

**Fig. 5:**
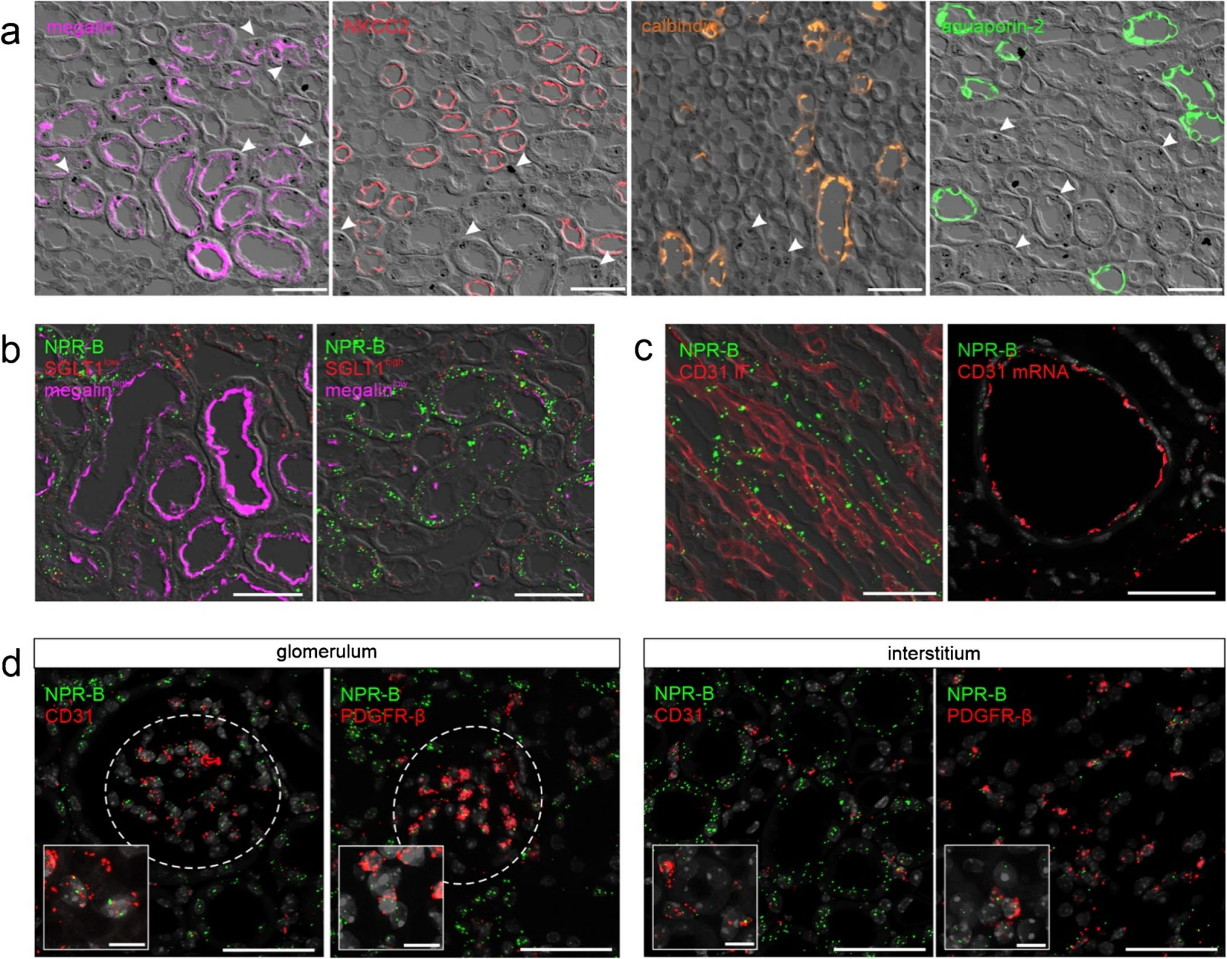
NPR-B mRNA expression in the murine kidney. **a** Chromogenic NPR-B RNAscope (black dots, arrows) followed by immunofluorescence staining of the different tubular segments: Megalin (PT, purple), Na–K-2Cl cotransporter (NKCC2, thick ascending loop of Henle, red), calbindin (distal tubule, orange), and aquaporin 2 (CD, green). **b** Multiplex in-situ hybridization of NPR-B (green) and the sodium-dependent glucose cotransporter 1 (SGLT1, red) plus megalin (purple) immunofluorescence staining. NPR-B mRNA was strongly detectable in the PT, with the highest expression in the S3 segment. **c** NPR-B mRNA is localized in vasa recta in renal medulla and papilla, but not in the endothelium of renal arterioles. **d** NPR-B mRNA signal (green) in glomeruli colocalized with mesangial (PDGFR-β) and endothelial (CD31) cell markers. Interstitial signals mainly derived from PDGFR-β^+^ fibroblasts but also from endothelial cells. Nuclei are counterstained with DAPI (grey). Glomeruli are visualized by white dashed lines. Scale bars are 50 µm, at higher magnification 10 µm

Strong NPR-B mRNA signal was detectable in tubular cells in renal cortex and medulla, while no NPR-B signal was visible in tubular cells in the papilla. Furthermore, NPR-B was expressed in some interstitial cells, in glomeruli and in straight structures between tubules in medulla and papilla, but with lower signal intensity compared with tubules. (Figs. 4 and 5c).

By conducting in situ hybridization followed by immunofluorescence staining of the individual tubular sections, we identified the proximal tubule (PT) as the NPR-B expressing nephron segment (Fig. 5a). Megalin expression in the PT decreases from S1 to S3, while the signal of the sodium-dependent glucose cotransporter 1 (SGLT1) increases from S1 to S3. NPR-B signal intensity increased along the PT and was the highest in SGLT1^high/^megalin^low^ S3 segments, mainly located in the medulla (Fig. 5b). In the thick ascending limb of the loop of Henle (NKCC2), distal tubules (calbindin), and collecting ducts (CD, aquaporin-2), only single NPR-B mRNA signals were found in a few tubules (Fig. 5a and b). Intrarenal arteries showed NPR-B signal, but to a lesser extent compared with NPR-A. There was no co-localization with the endothelial marker CD31, suggesting that the expressing cells are smooth muscle cells. The strongly NPR-B positive straight structures were identified as endothelial cells of vasa recta (Fig. 5c). Focusing on glomeruli and the interstitium, endothelial and PDGFR-β^+^ cells showed rather low NPR-B expression. In the medulla, most of the detected mRNA signal derived from endothelial cells, while in the papilla, PDGFR-β^+^ cells appeared as the main source of NPR-B expressing cells (Fig. 5d). In juxtaglomerular renin-producing cells, weak mRNA signal was found, while interstitial renin^+^ cells and epo-producing cells were not consistently NPR-B-positive (data not shown).

As visualized in RNAscope stainings, real-time PCR identified the S3 segment of the PT as a strong NPR-B expressing nephron segment of the murine kidney. Receptor expression was also detectable in the S1/S2 regions and the glomeruli, where the NPR-B signal is apparently not derived from podocytes (Fig. 3b).

The NPR-B mRNA signal was reduced in the kidneys of mice with adenine nephropathy in RNAscope and tended to be lower in real-time PCR data (Fig. 4 + 3e). Since NPR-B expression pattern was not altered in adenine nephropathy and the fibrotic areas in the adenine kidneys did not show increased NPR-B mRNA signals, no detailed investigation on receptor expression in immune cells was performed. A summary of NPR-B mRNA expression in murine kidneys is listed in Table 5.

**Table 5:** Overview of renal cell types expressing NPR-B mRNA in RNAscope in murine kidney

Nephron segments					Glomeruli				Interstitium				Vessels	
PT S1/S2	PT S3	TALH	DT	CD	Capillaries (CD31)	Mesangial cells (PDGFR-β)	Podocytes (nephrin)	JG renin cells	PDGFR-ß interstitial cells	Endothelial cells (CD31)	Epo-producing cells	Renin-producing cells	Endothelium (CD31)	Smooth muscle cells
+	** + + **	**-/ + **	**-/ + **	**-/ + **	** + **	** + **	**-**	** + **	** + **	** + **	**-/ + **	**-/ + **	**-**	** + **

Abbreviations: PT proximal tubule, TALH thick ascending loop of Henle, DT distal tubule, CD collecting duct, JG juxtaglomerular, epo erythropoietin.

Symbols representing expression levels:—= no expression. +  = (low) expression. +  +  = high expression.

### Expression of NPR-C in murine kidneys

**Fig. 6:**
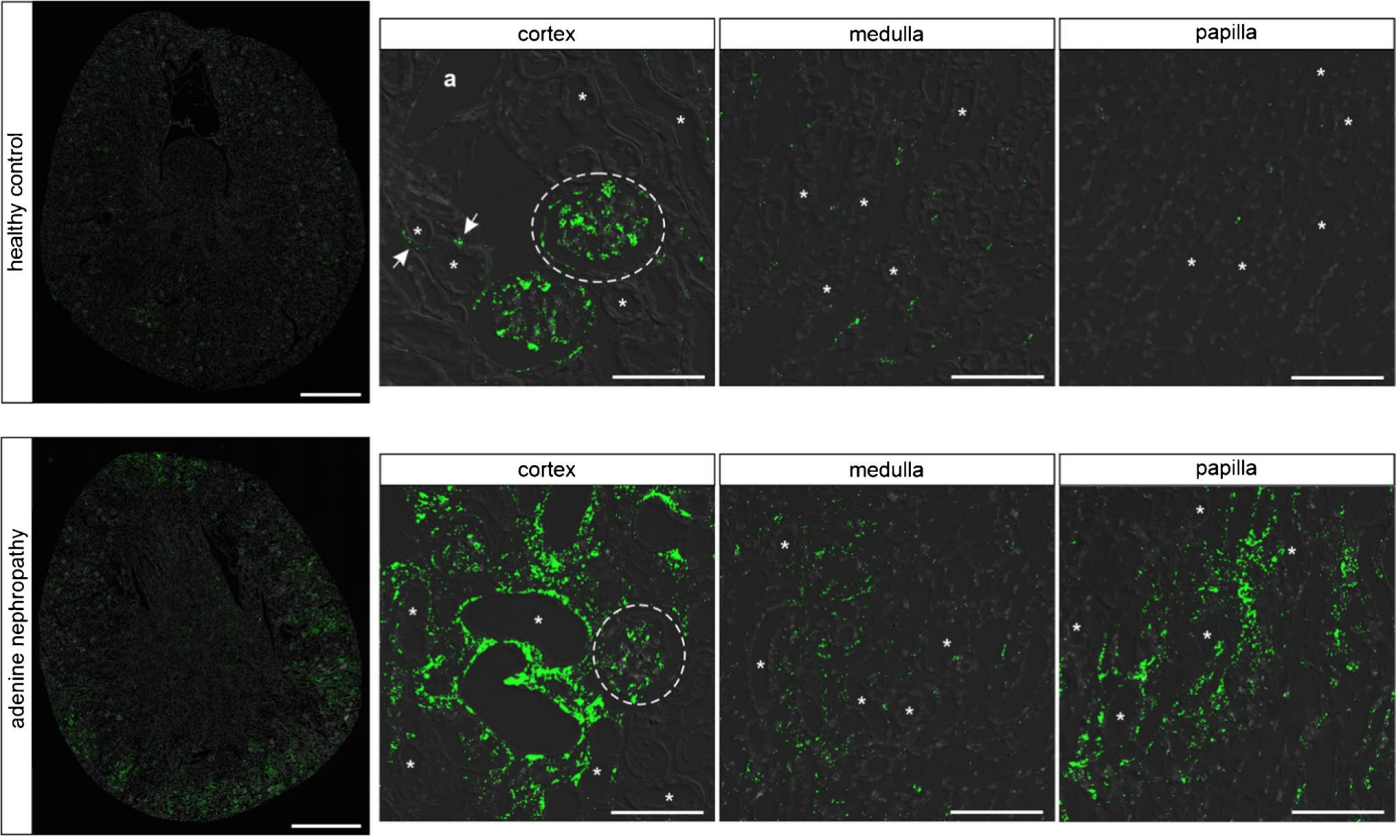
NPR-C mRNA expression in murine kidneys of control animals and in adenine nephropathy. NP clearance receptor NPR-C mRNA distribution (green) in healthy mouse kidneys and in adenine nephropathy. Under physiological conditions, only weak NPR-C expression was detectable in the mouse kidney, with the strongest signal in the glomeruli (white dashed lines). Additionally, NPR-C is also present in interstitial cells (marked with arrows). Renal tubules do not express NPR-C (marked with “*”). In adenine nephropathy, NPR-C signal was significantly increased and appeared mainly in cell clusters located in fibrotic areas, especially within the subcapsular cortex. Nuclei are counterstained with DAPI (grey). Overview scale bars are 1000 µm. Detail scale bars are 100 µm

The NPR-C plays a crucial role in the regulation of NP signaling due to its function as a clearance receptor for ANP, BNP, and CNP. Compared with NPR-A and -B, NPR-C mRNA expression was more restricted in healthy control animals, with the strongest signal in glomeruli. In addition, NPR-C mRNA was found sporadically in interstitial cells of the cortex and, to an even lower extent, in the medulla and papilla. As for NPR-A, the tubular system showed no NPR-C mRNA expression, neither did intrarenal blood vessels (Fig. 6).

**Fig. 7:**
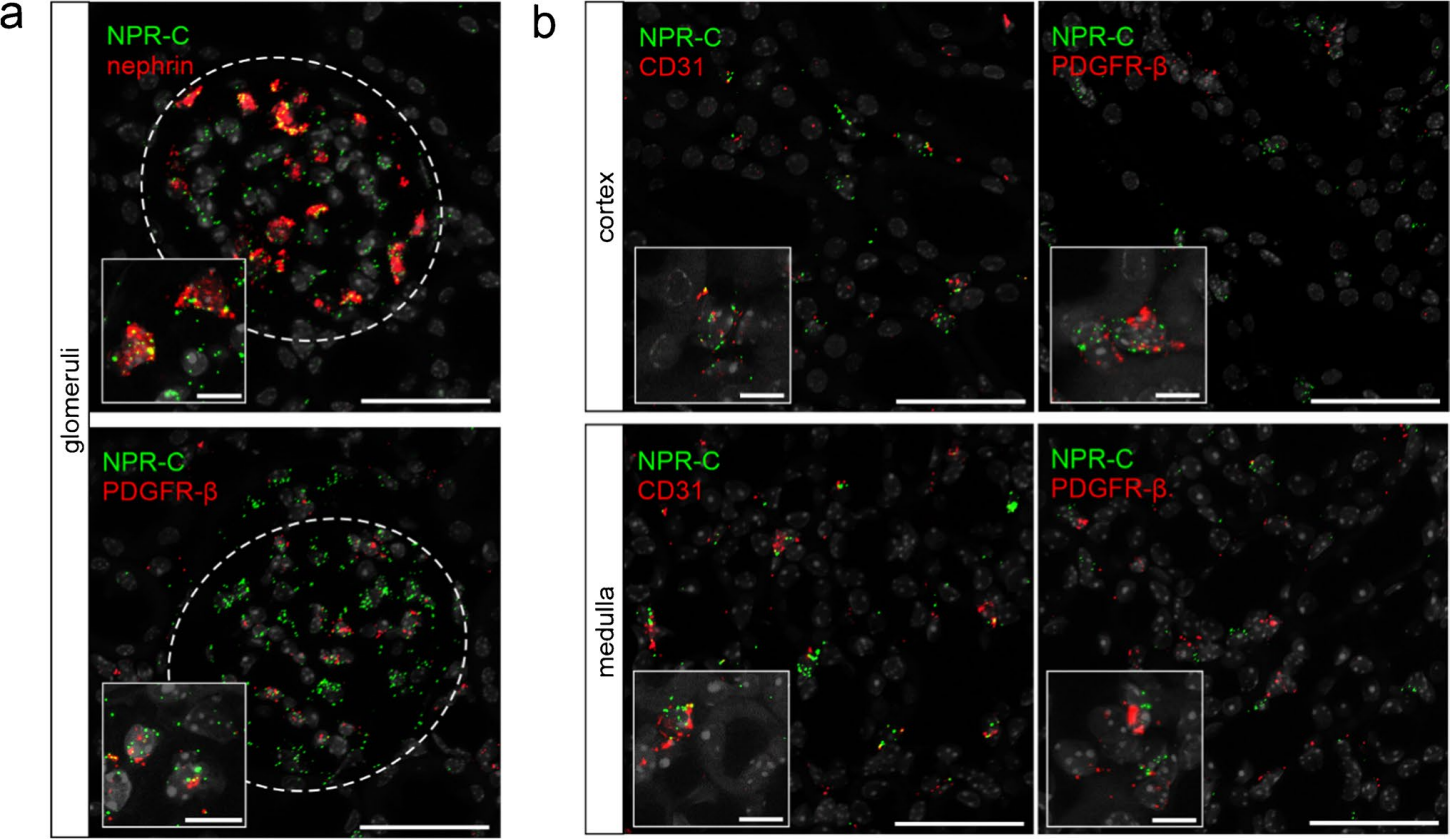
NPR-C mRNA expression in glomeruli and the interstitium of mouse kidneys. **a** NPR-B mRNA expression (green) in glomeruli is highly colocalized with nephrin (podocytes), and to a lower extend with PDGFR-β (mesangial cells). **b** Interstitial receptor distribution is relatively low, but some endothelial cells as well as single PDGFR-β-positive interstitial cells showed hybridization signal. Nuclei are counterstained with DAPI (grey). Glomeruli are visualized by white dashed lines. Scale bars are 50 µm, at higher magnification 10 µm

In the glomerulus, podocytes were identified to highly express NPR-C, but also mesangial cells showed a weak signal (Fig. 7a). NPR-C is also synthesized in capillaries and fibroblasts in the interstitium, where co-localization with CD31 was stronger than with PDGFR-β (Fig. 7b). Renin- and epo-producing cells did not show NPR-C mRNA signal (not shown). Realtime PCR largely confirmed in-situ hybridization data since NPR-C mRNA expression was most abundant in FACS sorted podocytes (Fig. 3c). However, PCR further detected weak NPR-C expression in the tubular system, where expression levels were similar between the different segments but much lower compared to glomeruli.

**Fig. 8:**
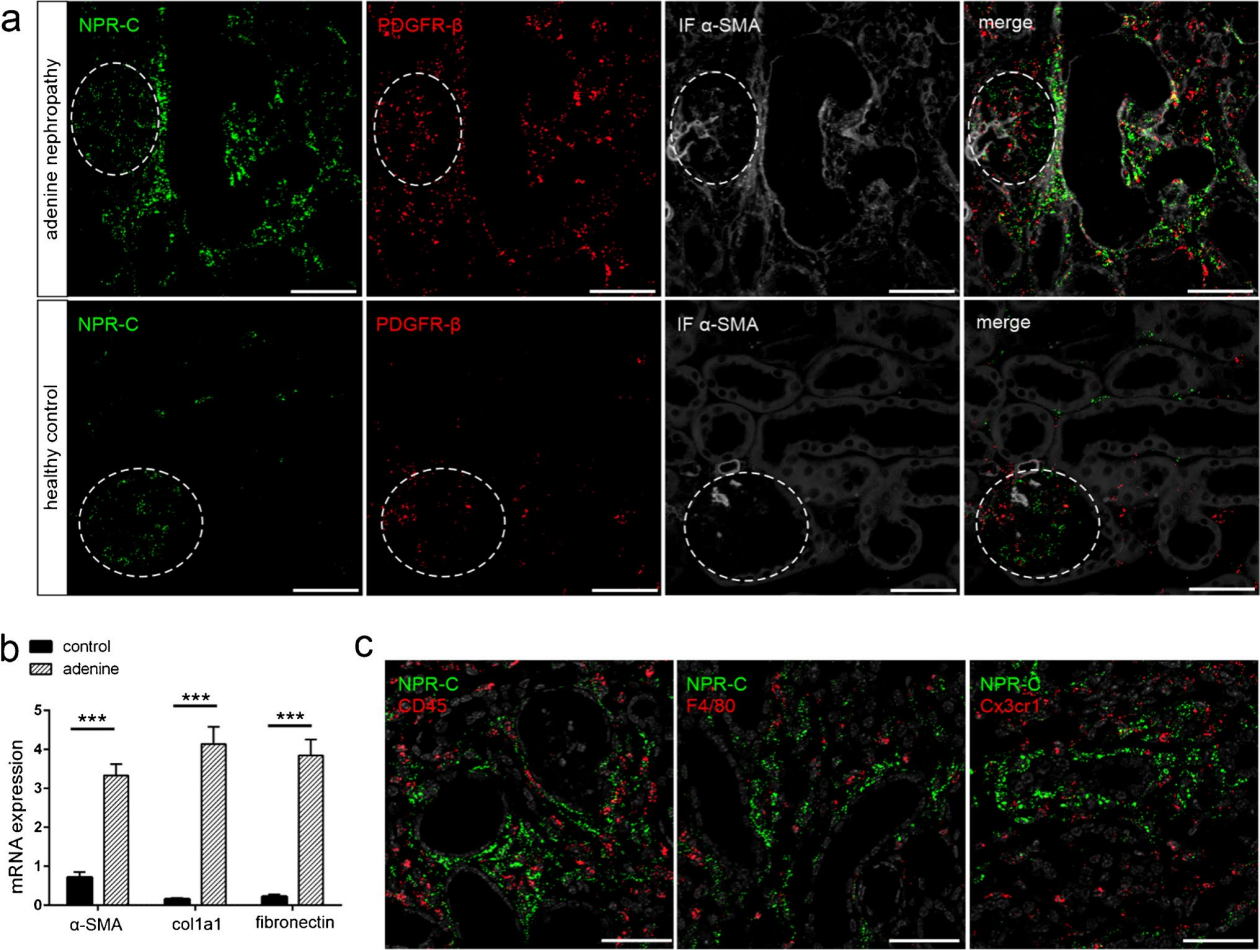
NPR-C mRNA expression in adenine nephropathy. Investigation of highly NPR-C mRNA expressing (green) cell types in kidneys with adenine nephropathy. **a** Most NPR-C signal derives from PDGFR-β-and α-SMA-positive interstitial myofibroblasts in fibrotic areas. **b** Fibrotic changes in adenine nephropathy shown by the significantly increased production of the extracellular matrix proteins α-SMA, collagen 1a1 and fibronectin. n(control) = 4, n(adenine) = 6. **c** Leukocytes, macrophages, and dendritic cells showed no NPR-C signal, but immune cells were located within the areas with the highest receptor expression. Nuclei are counterstained with DAPI (grey). Glomeruli are visualized by white dashed lines. Scale bars are 50 µm. Statistical significance is indicated by *p < 0.05, **p < 0.01, and ***p < 0.001

RNAscope in kidneys with adenine nephropathy showed a marked increase in NPR-C mRNA expression in cell clusters within fibrotic areas, especially in the subcapsular region, but also in the medulla and papilla, where only little expression was found in healthy animals (Fig. 6). PDGFR-β and α-SMA co-expressing myofibroblasts in the renal interstitium were identified as the main NPR-C expressing cell population in adenine nephropathy (Fig. 8a). Adenine-induced fibrotic remodelling of renal tissue is shown by significantly increased production of extracellular matrix proteins α-SMA, collagen 1a1 and fibronectin (Fig. 8b). Macrophages, DCs and leukocytes showed no NPR-C signal. However, it was noticeable that the immune cells were mostly located in the immediate vicinity of highly NPR-C expressing cells within fibrotic areas (Fig. 8c). In contrast to the visual impression, semi-quantitative realtime PCR revealed no increase of NPR-C mRNA expression in adenine kidneys but rather indicated decreasing receptor mRNA levels under inflammatory conditions (Fig. 3f).

In summary, under control conditions, NPR-C was primarily found in podocytes of the glomeruli, but also in mesangial, endothelial, and PDGFR-β^+^ interstitial cells. In mice with adenine nephropathy, there was a massive increase in NPR-C expression, particularly in fibrotic areas in the subcapsular cortex, which was mainly derived from PDGFR-β^+^ myofibroblasts. No NPR-C mRNA signal was detectable in immune cells. An overview of NPR-C distribution is listed in Table 6.

**Table 6:** Overview of renal cell types expressing NPR-C mRNA in RNAscope in healthy and diseased murine kidney

Glomeruli				Interstitium			Vessels	Inflammation (adenine nephropathy)				
Capillaries (CD31)	Mesangial cells (PDGFR-β)	Podocytes (nephrin)	JG renin cells	PDGFR-ß interstitial cells	Endothelial cells (CD31)	Renin/epo producing cells		PDGFR-ß interstitial cells	Capillaries (CD31)	Leukocytes (CD45)	Macrophages (F4/80)	Dendritic cells (Cx3cr1)
-	** + **	** + + **	**-**	** + **	** + + **	**-**	**-**	** + + **	** + **	**-**	**-**	**-**

Symbols representing expression levels:—= no expression. +  = (low) expression. +  +  = high expression.

## Discussion

The NPR-A receptor (guanylyl cyclase A), which mediates the effects of ANP and BNP, has a broad tissue distribution in murine kidneys, since it is expressed in glomeruli, intrarenal arterioles, renin- and epo-producing cells, PDGFR-β-positive interstitial cells, and endothelial cells of peritubular capillaries and vasa recta. Unexpectedly, but in line with results in human kidneys, no NPR-A mRNA signal was detected by RNAscope in the tubular system [19]. This result, which is in contrast to previous expression studies and functional studies showing direct effects of ANP on tubular cells (for review see [69]), might be explained by expression levels below the limit of detection by RNAscope. In fact, realtime PCR of microdissected nephron segments revealed NPR-A mRNA expression in tubules, especially in collecting ducts that, however, was very low compared with NPR-A expression in glomeruli (Fig. 3). It should be mentioned that the weak NPR-A expression in isolated tubules could be due to contamination with adherent interstitial cells and therefore does not necessarily prove expression in tubular cells. In summary, our data indicate that, if present at all, NPR-A mRNA expression in tubular cells is very low, calling into question a direct tubular effect of ANP in vivo. Further functional studies in tubule-specific knockout mice may clarify this issue. While our RNAscope data do not definitely exclude that tubular cells express NPR-A, it direct special interest to those cell types with high NPR-A expression. First and foremost is the prominent expression of NPR-A in podocytes that has already been described in different species including humans [19, 64]. Podocyte-restricted deletion of NPR-A did not attenuate the effects of ANP and BNP on kidney perfusion, GFR and natriuresis but markedly aggravated glomerular damage in the hypervolemic UNx/DOCA-salt model, indicating a direct protective effect of NP/NPR-A signaling [64]. Vascular smooth muscle cells of renal arterioles of all diameters, including afferent and efferent arterioles, express NPR-A which is the basis for ANP mediated increase in renal perfusion and GFR. Early studies indicated that ANP dilates afferent arterioles while it constricts efferent arterioles, hereby increasing intraglomerular filtration pressure [38]. Moreover, renin-producing cells in afferent arterioles express NPR-A, which is a prerequisite for direct inhibition of renin release by ANP [35]. Recently, renal interstitial cells have been shown to express renin mRNA, without a detailed characterization of the regulation and function of interstitial renin [3]. Our data show expression of NPR-A in these interstitial renin-producing cells, suggesting that ANP and BNP may have a regulatory role here. In addition, epo-producing interstitial cells express NPR-A. Acute infusion of ANP resulted in a rapid increase of hematocrit, which, however, was due to ANP-mediated intravascular volume contraction and cannot be induced by epo-dependent stimulation of hematopoiesis in the short observational period [53]. Evidence for an influence of ANP or BNP on epo formation and subsequent changes in hematocrit is not yet available, and the fact that NPR-A knockout mice have normal hematocrit levels under control conditions argues against this relationship [36, 56]. However, a possible role of NPs in pathologic conditions, in which ANP and BNP concentrations increase significantly, should be considered. Of note, the BNP promotor contains a hypoxia response element which is activated by the hypoxia inducible factor-1 (HIF1) and hypoxia markedly stimulates BNP mRNA expression in cardiomyocytes, so that a link between hypoxia, BNP and epo might exist [73]. In addition to podocytes, we found the highest NPR-A expression in endothelial cells of peritubular capillaries and vasa recta. In agreement, it was shown previously, that selective deletion of NPR-A in endothelial cells reduces total NPR-A mRNA abundance in the kidney by more than 50% [53]. Activation of NPR-A increases endothelial permeability resulting in extravasation of albumin and an accompanying fluid shift into the interstitium [53]. Accordingly, mice with endothelium-specific deletion of NPR-A have hypervolemic hypertension. Under control conditions urine and sodium excretion of these mice are not significantly different compared with control mice, but more detailed analyses of kidney function under challenging conditions were not performed [53]. Accordingly, the biological function of renal endothelial NPR-A remains unclear. It might be speculated that ANP-mediated plasma extravasation, which occurs in several organs including the kidney, changes osmotic driving forces in the renal tubulointerstitium and affects tubular reabsorption [53]. Moreover, endothelium-derived relaxation of vasa recta could increase medullary perfusion, resulting in a washout of interstitial osmolytes and consequently reduced water reabsorption, a well-known effect of NPs. Finally, ANP attenuates inflammatory activation of endothelial cells and expression of adhesion molecules, hereby controlling extravasation of immune cells [20, 71]. Since endothelial cells are considered mediators of renal inflammation, ANP/BNP might exert renoprotection via endothelial NPR-A activation [45]. Finally, significant NPR-A expression was detected in PDGFR-β-positive interstitial cells that include pericytes, fibroblasts, and epo-producing cells (see above). Pericytes are regulators of vasa recta tone and renal medullary perfusion [29]. Deletion of NPR-A in PDGFR-β-positive cells completely abolishes the vasodilating effects of ANP on capillaries in the cremaster muscle and the retinal microcirculation and results in arterial hypertension [63]. While no alterations of urine and sodium excretion under control conditions were found in vivo, the ANP-induced increase in renal perfusion of isolated perfused kidneys was significantly attenuated in mice with PDGFR-β-cell specific deletion of NPR-A. Noteworthy, this effect was only observed in kidneys in which blood vessels had been pre-constricted with endothelin-1 and not in untreated control kidneys [63]. Thus, further investigations on the role of NPR-A in renal PDGFR-β-positive cells are needed.

Several studies have already demonstrated the nephroprotective properties of NPs, which are at least partially independent of their hypotensive and RAAS antagonizing effects [11, 44, 64]. To test for a possible regulation of NPRs in kidney disease, we examined their distribution in adenine nephropathy. While RNAscope signals of NPR-A and -B were markedly reduced in adenine nephropathy, their spatial distribution remained unaltered. It is unclear whether the downregulation of NPR-A and -B occurs as a direct physiological response to adenine-induced processes or as a negative feedback to increased NP levels seen in cardiorenal disease [54]. Only little NPR-A and -B mRNA signal was detectable in the fibrotic areas heavily infiltrated by immune cells and co-hybridization with immune cell-specific markers did not show any overlap. These observations do not support the hypothesis of a direct effect of NPs on these cells, but rather suggest that the previously observed high levels of proinflammatory cytokines in kidneys of NPR-A knockout mice indirectly result from hypertensive kidney damage in this mouse strain [21, 70]. Since NPR expression was shown previously in different cell types of the innate and adaptive immune system of humans and rodents [5, 15, 30, 43], it is also possible that the expression level was below the detection limit of RNAscope in our study.

RNAscope for NPR-B (guanylyl cyclase B), which is the receptor for CNP, showed significant overlap with NPR-A, although at different signal intensities. This was shown for vascular smooth muscle cells, endothelial cells of vasa recta and PDGFR-β-positive cells. Since both receptors are membrane-bound guanylyl cyclases synthesizing cGMP, similar cellular effects can be assumed upon stimulation. In fact, similar to NPR-A, cell specific deletion of NPR-B in PDGFR-β-positive cells prevented the dilatory effects of CNP on pre-capillary arterioles and capillaries of the cremaster muscle and the retinal circulation and induced arterial hypertension [62, 63]. Urine and sodium excretion was unaltered under control conditions in these mice [62]. The endowment of the aforementioned cells with two NP receptor types that activate the same intracellular signaling cascade, appears redundant at first glance. However, the activating hormones differ considerably in their characteristics. While ANP and BNP are classical circulating hormones whose release is stimulated for instance by cardiac wall tension, CNP is a paracrine hormone from different sources which is regulated by completely different factors. Besides the endothelium, CNP is synthesized in the kidney. Since renal CNP is low under normal conditions but is elevated in several form of renal diseases such as nephrotic syndrome, unilateral ureteral obstruction or diabetes mellitus in patients or rodent models, studies on the functional role of CNP and NPR-B mainly focused on disease conditions [7, 25, 50, 61].

In contrast to NPR-A, RNAscope as well as real-time PCR results showed that murine NPR-B is markedly expressed in proximal tubules, with the strongest signal in the S3 segment. Moreover, strong expression was found in the vasa recta. The presence of NPR-B in the murine vasa recta has already been reported by other groups [68]. In contrast, the detection of NPR-B mRNA in the tubular system is surprising, as CNP lacks natriuretic and diuretic potential in experimental animals and healthy volunteers at least at physiological concentrations [10, 12, 49]. Moreover, this observation contrasts the experiments conducted in human tissue, where no renal NPR-B mRNA expression was detectable in tubular cells [19]. While the RNAscope probe used in our experiments covers all NPR-B protein-coding variants, the one available for human tissue does not detect at least one alternative protein-coding mRNA construct starting with exon 13 [19]. By using primers binding exon 18/exon 19, Hirsch et al. detected NPR-B expression in human tubules. Further, they reported variant-dependent expression of the receptor and located normal NPR-B in the glomeruli and proximal tubules, which agrees with our observations [22]. CNP has been shown to be highly protective on glomerular health by mediating anti-fibrotic and anti-proliferative processes, such as inhibition of mesangial cell proliferation and of the accumulation of extracellular matrix proteins [6]. It is also possible that the controversial results regarding NPR-B expression are due to species-dependent differences. Experiments with labeled CNP and immunohistochemical stainings have identified binding sites in renal tissue whose localization is also consistent with our NPR-B in-situ hybridization results [16, 75].

NPR-B expression in the PT suggests CNP as a regulator of tubular transport in this nephron segment, which is compatible with previous results in NPR-B knockout mice that have impaired diuresis and developed salt-dependent renal injury [28]. Because of the nephroprotective potential of CNP, observed in various animal experiments, the expression of NPR-B in the PT might exert a primary protective function [9, 28, 77]. Interestingly, marked protective effects of CNP treatment was observed in models with primary damage of proximal tubules, such as cisplatin-induced nephrotoxicity, ischemia/reperfusion or hemorrhagic shock [8, 28, 31]. The PT has enormous transport activity and therefore is particularly susceptible to damage. Especially in the S3-segment a disproportion between oxygen consumption and supply exists. Accordingly, this segment is usually particularly vulnerable, especially in the case of reduced blood supply [60]. It would be conceivable that expression of the NPR-B receptor in this nephron segment primarily serves nephroprotection.

Under physiological conditions, the expression of NPR-C is relatively restricted in murine kidneys. It is responsible for the degradation of circulating NPs but may also exert biological effects itself, mediated mainly through the adenylate cyclase/cAMP signaling pathway. Instead of an intracellular guanylylcyclase, NPR-C receptor is presumably coupled to a pertussis toxin-sensitive inhibitory G protein domain [52]. In our study, the strongest expression of NPR-C in the kidney occurred in podocytes, where also NPR-A mRNA is highly concentrated, followed by endothelial cells. The presence of NPR-C in podocytes has already been described before [64]. Real-time PCR revealed low receptor expression in the tubular system. As discussed for NPR-A, the different detection limits of RNAscope technique and PCR or contamination of microdissected tubules with adherent cells may be the explanation for the inconsistent results.

In adenine nephropathy, we observed a strong increase of NPR-C mRNA signal, which was mainly derived from PDGFR-β-positive myofibroblasts in fibrotic areas. It has already been shown that stimulation of renal PDGFR-β induces tubulointerstitial cell proliferation, formation of myofibroblasts, and fibrotic tissue remodeling under disease conditions [18]. This observation makes the strong expression of the NPR-C receptor in fibrotic areas interesting. On the one hand, the NPR-C receptor could act in its classical function as NP clearance receptor under the given inflammatory conditions. In doing so, it would degrade circulating and locally produced NPs and thereby attenuate their effects on the target cells. In the tubulointerstitial damage model of the unilateral ureteral obstruction (UUO), in addition to tubular cells, interstitial myofibroblasts synthesize CNP [66]. On the other hand, NPR-C might directly activate intracellular signaling as a negative regulator of the adenylate cyclase/cAMP system [26]. Since the cAMP pathway induces anti-fibrotic processes, the NPR-C-induced reduction of cAMP levels would promote fibrosis in this case [55]. In contrast to this possibility, NPR-C signaling is considered to promote anti-proliferative and -inflammatory processes [26, 52, 58]. Further, the receptor appeared to have a protective role in ischemia/reperfusion injury model [23]. An increase in NPR-C receptor expression in renal and cardiovascular disease is already reported in the literature [34, 52], but comparing our realtime PCR data with the results of in situ hybridization, the increased expression of the NPR-C receptor in the fibrotic areas is not evident at the whole organ level. This discrepancy may be a consequence from the upregulation of NPR-C in myofibroblasts on the one hand and a downregulation of NPR-C in other cells in adenine nephropathy, which compensates high NPR-C expression in myofibroblasts at the whole organ level.

In conclusion, all NPRs are expressed in murine renal tissue and are differentially regulated in renal injury. By identifying new NPR expressing cells types in the kidney, this work provides the basis for further investigations on the renal effects of natriuretic peptides in health and disease.

## Supplementary information

Below is the link to the electronic supplementary material.

Supplementary file1 (DOCX 4689 KB)

## Data Availability

The datasets generated during the current study are available from the corresponding author on reasonable request.
