# Supplementary material for: Localization of natriuretic peptide receptors A, B, and C in healthy and diseased mouse kidneys
Source: Pflugers Arch. 2022 Dec 8;475(3):343–60. doi: 10.1007/s00424-022-02774-9 (PMC9908653; doi:10.1007/s00424-022-02774-9)
Supplement: Supplementary file 1 — Supplementary file1 (DOCX 4689 KB) [file 424_2022_2774_MOESM1_ESM.docx]

## Supplementary Information (SI)

For the article

“Localization of natriuretic peptide receptors A, B and C in in healthy and diseased mouse kidneys”

Authors:

Elena-Sofia Heinl, Katharina Anna-Elisabeth Broeker, Claudia Lehrmann, Rosmarie Heydn, Katharina Krieger, Katharina Ortmaier, Philipp Tauber and Frank Schweda

Institute for Physiology, University Regensburg, Germany

Corresponding Authors:

Elena-Sofia Heinl and Frank Schweda

Institute for Physiology, University Regensburg, Germany

Email: elena.heinl@ur.de; frank.schweda@ukr.de

Submitted in Pflügers Archiv - European Journal of Physiology


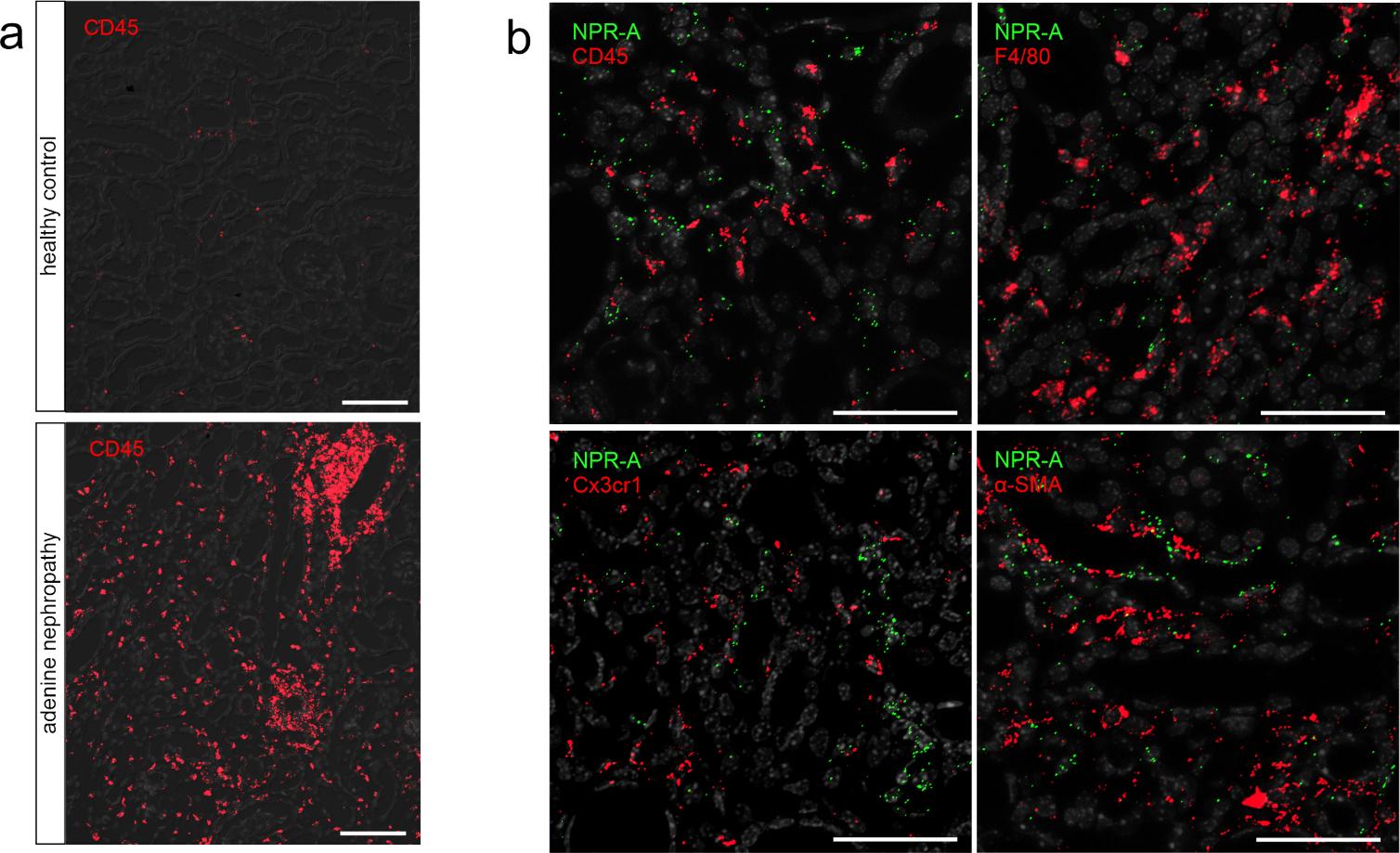


**Online Resource 1** **NPR-A mRNA expression of inflammatory cells in adenine nephropathy**

NPR-A mRNA expression (green) in various immune cells (red) infiltrating kidneys in adenine nephropathy. (**a**) Kidneys of healthy and adenine-treated mice. Adenine-feeding induced massive infiltration of immune cells (red) and fibrosis. Scale bars are 100 µm (**b**) No robust NPR-A expression in lymphocytes (CD45), macrophages (F4/80), DCs (Cx3cr1) and myofibroblasts (α-SMA). Nuclei are counterstained with DAPI (grey). Scale bars are 50 µm.


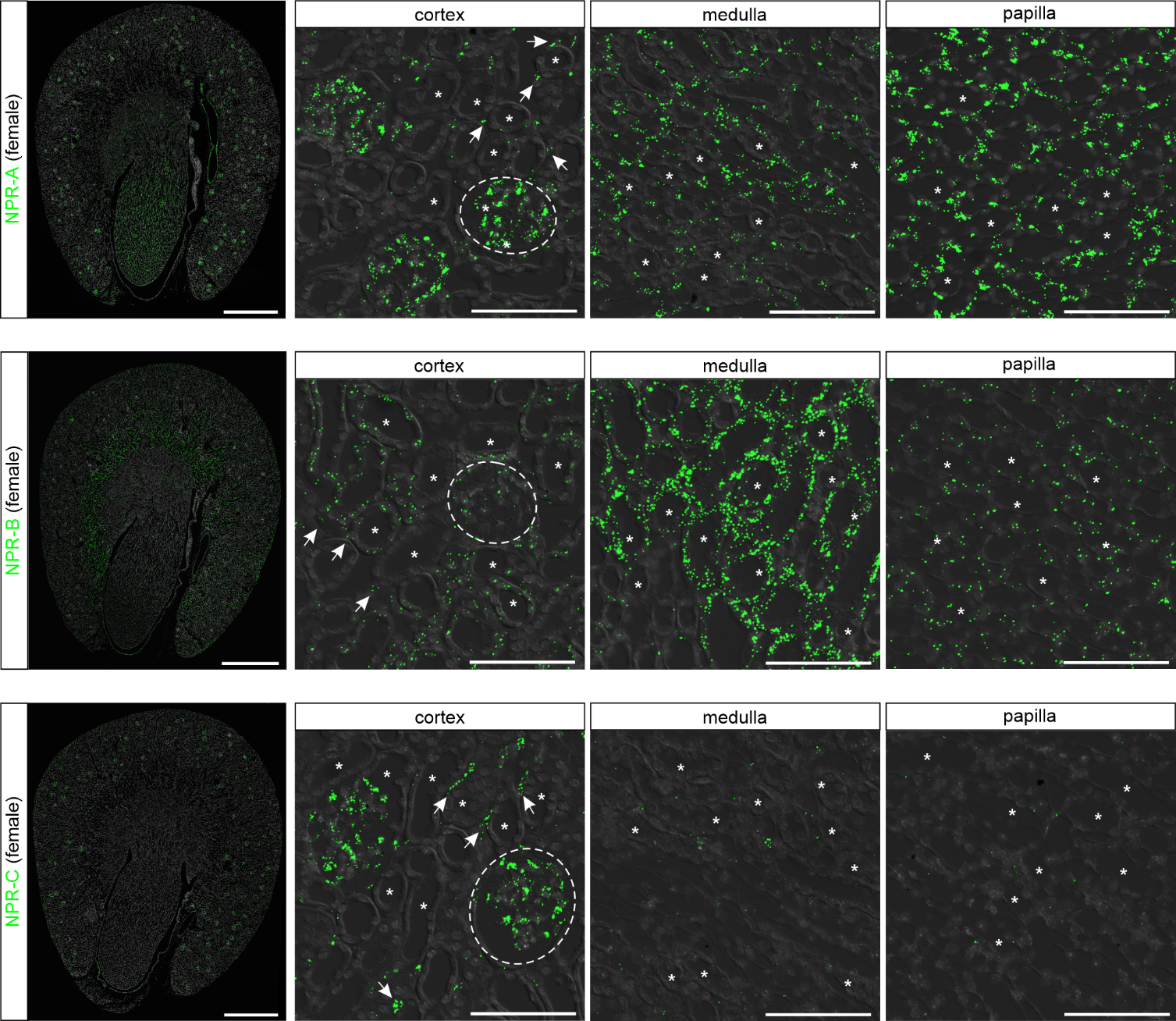


**Online Resource 2 NPR-A, NPR-B and NPR-C mRNA expression in kidneys of female mice under physiological conditions**

The localization of NPR-A, NPR-B, and NPR-C mRNA in kidneys of females is consistent with the expression pattern described in males (figures 1, 4, 6). NPR-A mRNA is mainly expressed in glomeruli, renal arterioles, and interstitial cells while no mRNA expression was detectable in tubules. The NPR-B receptor is expressed in the cortical and medullary tubular system and, at lower levels, in interstitial cells and glomeruli. NPR-C mRNA was highly detectable in the glomeruli and is also expressed in interstitial cells but not in renal tubules. Glomeruli are visualized by white dashed lines, tubules are marked as “*” and interstitial cells are indicated by white arrows. Nuclei are counterstained with DAPI (grey). Overview scale bars are 1000 µm. Detail scale bars are 100 µm.


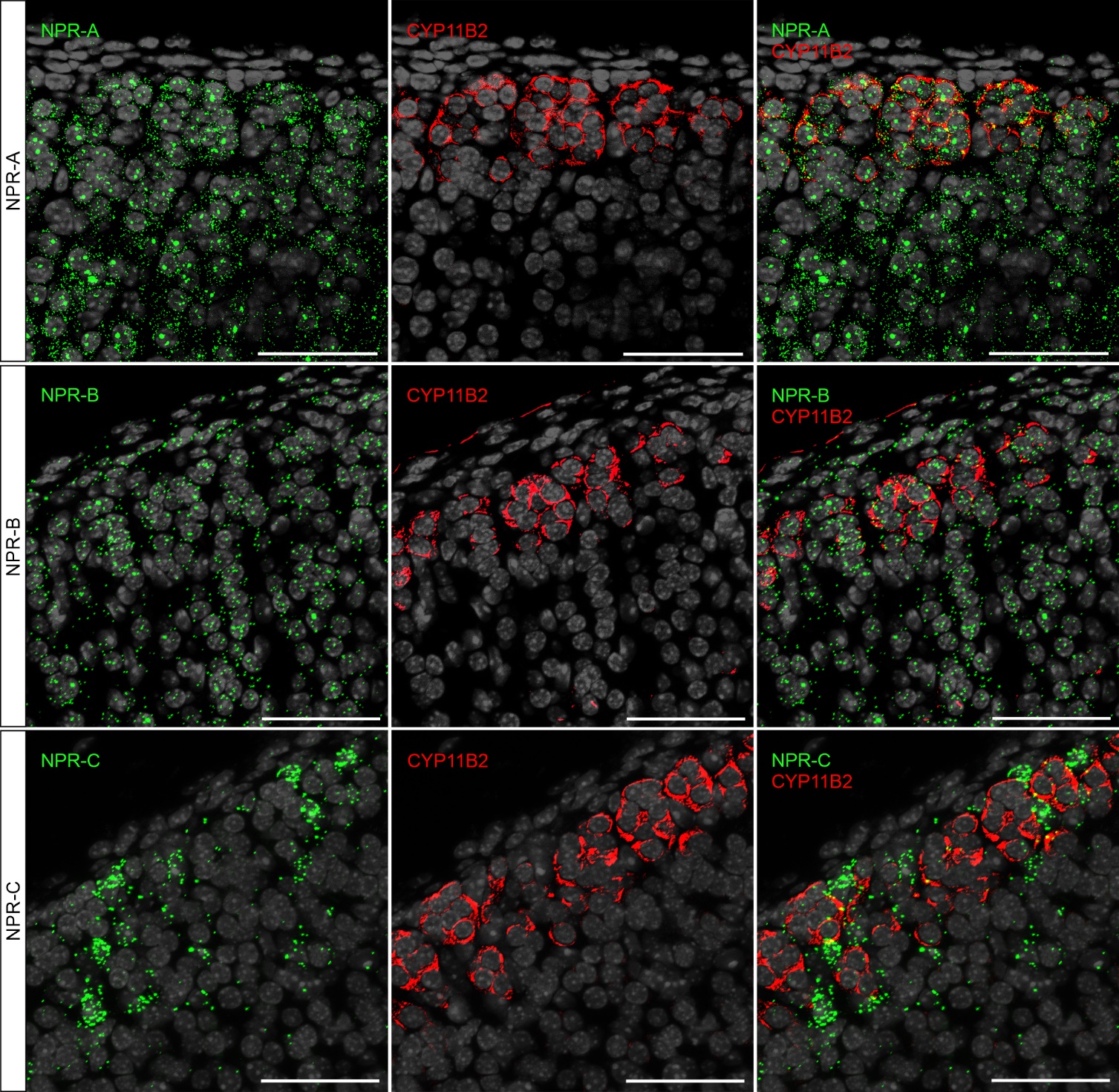


**Online Resource 3 NPR-A, NPR-B and NPR-C mRNA expression in aldosterone-producing cells of murine adrenal glands**

In situ hybridization of NPR-A, NPR-B, and NPR-C (green) in mouse adrenal glands. Aldosterone-producing cells were identified by immunofluorescence staining of CYP11B2 (aldosterone synthase, red). mRNA of all three receptors is expressed in the zona glomerulosa of murine adrenal glands. NPR-A and, to a lesser extent, NPR-B expression was detectable in aldosterone-producing cells. NPR-C was more restricted and less abundant in, but rather highly concentrated between cells positively stained for CYP11B2. Nuclei are counterstained with DAPI (grey). Scale bars are 50 µm.
